# Author Correction: Asymmetric thinning of the cerebral cortex across the adult lifespan is accelerated in Alzheimer’s disease

**DOI:** 10.1038/s41467-022-28514-2

**Published:** 2022-02-07

**Authors:** James M. Roe, Didac Vidal-Piñeiro, Øystein Sørensen, Andreas M. Brandmaier, Sandra Düzel, Hector A. Gonzalez, Rogier A. Kievit, Ethan Knights, Simone Kühn, Ulman Lindenberger, Athanasia M. Mowinckel, Lars Nyberg, Denise C. Park, Sara Pudas, Melissa M. Rundle, Kristine B. Walhovd, Anders M. Fjell, René Westerhausen, The Australian Imaging Biomarkers and Lifestyle Flagship Study of Ageing, Colin L. Masters, Ashley I. Bush, Christopher Fowler, David Darby, Kelly Pertile, Carolina Restrepo, Blaine Roberts, Jo Robertson, Rebecca Rumble, Tim Ryan, Steven Collins, Christine Thai, Brett Trounson, Kate Lennon, Qiao-Xin Li, Fernanda Yevenes Ugarte, Irene Volitakis, Michael Vovos, Rob Williams, Jenalle Baker, Alyce Russell, Madeline Peretti, Lidija Milicic, Lucy Lim, Mark Rodrigues, Kevin Taddei, Tania Taddei, Eugene Hone, Florence Lim, Shane Fernandez, Stephanie Rainey-Smith, Steve Pedrini, Ralph Martins, James Doecke, Pierrick Bourgeat, Jurgen Fripp, Simon Gibson, Hugo Leroux, David Hanson, Vincent Dore, Ping Zhang, Samantha Burnham, Christopher C. Rowe, Victor L. Villemagne, Paul Yates, Sveltana Bozin Pejoska, Gareth Jones, David Ames, Elizabeth Cyarto, Nicola Lautenschlager, Kevin Barnham, Lesley Cheng, Andy Hill, Neil Killeen, Paul Maruff, Brendan Silbert, Belinda Brown, Harmid Sohrabi, Greg Savage, Michael Vacher

**Affiliations:** 1 Center for Lifespan Changes in Brain and Cognition, Department of Psychology, University of Oslo, Oslo, Norway; 2 Center for Lifespan Psychology, Max Planck Institute for Human Development, Berlin, Germany; 3 Center for Vital Longevity, University of Texas, Dallas, TX USA; 4 MRC Cognition and Brain Sciences Unit, University of Cambridge, Cambridge, UK; 5 Department of Psychiatry and Psychotherapy, University Medical Center Hamburg-Eppendorf, Hamburg, Germany; 6 Max Planck UCL Centre for Computational Psychiatry and Ageing Research, Berlin, Germany; 7 Umeå Center for Functional Brain Imaging and Department of Integrative Medical Biology, Umeå University, Umeå, Sweden; 8 Department of Radiology and Nuclear Medicine, Oslo University Hospital, Oslo, Norway; 9 The Florey Institute, The University of Melbourne, Parkville, VIC Australia; 10 Collaborative Genomics Group, Centre of Excellence for Alzheimer’s Disease Research and Care, School of Medical and Health Sciences, Edith Cowan University, Joondalup, WA Australia; 11 School of Medical and Health Sciences, Edith Cowan University, Joondalup, WA Australia; 12 CSIRO, Herston, QLD Australia; 13 CSIRO, Melbourne, VIC Australia; 14 Department of Molecular Imaging, Austin Health, Heidelberg, VIC Australia; 15 National Ageing Research Institute, Parkville, VIC Australia; 16 Bio21 Institute of Molecular Science and Biotechnology, The University of Melbourne, Parkville, VIC Australia; 17 The University of Melbourne, Parkville, VIC Australia; 18 Cogstate Ltd., Melbourne, VIC Australia; 19 St. Vincent Hospital, Fitzroy, VIC Australia; 20 Department of Exercise Science, College of Science, Health, Engineering and Education, Murdoch University, Murdoch, WA Australia; 21 Department of Psychology, Macquarie University, Sydney, NSW Australia; 22 CSIRO, Floreat, WA Australia

**Keywords:** Cognitive ageing, Alzheimer's disease, Neural ageing

Correction to: *Nature Communications* 10.1038/s41467-021-21057-y, published online 1 February 2021.

In this article the affiliation details for Ulman Lindenberger were incorrectly given as ‘Department of Psychiatry and Psychotherapy, University Medical Center Hamburg-Eppendorf, Hamburg, Germany’ but should have been ‘Center for Lifespan Psychology, Max Planck Institute for Human Development, Berlin, Germany’, ‘Max Planck UCL Centre for Computational Psychiatry and Ageing Research, Berlin, Germany’. The original article has been corrected.

