# Supplementary material for: Asymmetric thinning of the cerebral cortex across the adult lifespan is accelerated in Alzheimer’s disease
Source: Nat Commun. 2021 Feb 1;12:721. doi: 10.1038/s41467-021-21057-y (PMC7851164; doi:10.1038/s41467-021-21057-y)
Supplement: Supplementary file 1 — Supplementary Information [file 41467_2021_21057_MOESM1_ESM.pdf]

Asymmetric thinning of the cerebral cortex across the adult lifespan is accelerated in Alzheimer's Disease

Roe et al.

Supplementary Information

Supplementary Methods......1

Supplementary Tables 1-3 5

Supplementary Fig. 1......6

Supplementary Note 1......7

Supplementary Fig. 2......7

Supplementary Fig. 3......8

Supplementary Fig. 4......9

Supplementary Fig. 5......10

Supplementary Note 2......10

Supplementary Fig. 6......11

Supplementary Fig. 7......12

Supplementary Fig. 8......13

Supplementary Fig. 9......13-15

Supplementary Fig. 10......16

Supplementary Table 4......16

Supplementary Table 5......16

Supplementary Note 3......17

Supplementary Table 6......17

Supplementary Note 4......18

Supplementary References......19

Supplementary Methods

Samples. The main discovery sample consisted of magnetic resonance imaging (MRI) data collected across 5 projects at the Center for Lifespan Changes in Brain and Cognition (LCBC; Department of Psychology, University of Oslo): Neurocognitive Development [1]; Cognition and Plasticity Through the Lifespan [2]; Constructive Memory [3]; Method of Loci [4]; and Neurocognitive Plasticity [5]. Because the latter two studies involved cognitive training, only MRI data from these projects was considered (see below description for each LCBC sub-study included here). For 563 individuals, more than one scan was available; 1851 of these were longitudinal in nature (mean number of longitudinal scans per participant = 3.3), whereas 205 were double-scanned at the same timepoint on both LCBC scanners (1.5T Avanto and 3T Skyra; Siemens Medical Solutions). The number of timepoints ranged from 1 to 6 (mean = 3.3 ± 1.8) and the max follow-up interval was 11.0 years since initial scan. For cognitive analyses, cognitive testing was conducted on average 19.0 days post-scan and all were tested within 5 months.

Below, we detail the main characteristics and participants of each LCBC sub-study, as also described in [6]. Common exclusion criteria across all LCBC projects are outlined in the main paper. **Neurocognitive Development.** This study consists of a longitudinal sample of children and adolescents acquired at the center for LCBC. Typically developing children and adolescents aged between 8 and 19 years were initially recruited through newspaper ads and local schools. Written informed consent was obtained from all participants older than 12 years of age and from a parent of participants under 16 years of age. Oral informed consent was given by participants under 12 years of age. The study was approved by the Regional Ethical Committee of South Norway. In addition to the exclusion criteria common to all LCBC studies detailed in the main paper, participants were required not have had a complicated or premature birth. Data acquisition took place between 2007 and 2016. The whole study consists of three waves, though only data from waves 2 and 3 were included in the present paper due to the 20+ age-range. MRI sequences were acquired either with a 1.5T Avanto or a 3.0T Skyra scanner (Supplementary Table 3). See [1,7] for more details. 179 observations from 103 participants (mean age = 22.4; females = 66; nTimepoints = 1-2) were included in the main LCBC sample here. **Cognition and Plasticity through the Lifespan**. This study is a longitudinal study where cognitively healthy adults underwent MRI scanning and neuropsychological evaluation. The sample was collected at the center for LCBC. Volunteers were initially recruited by newspaper advertisements and later contacted by mail for follow-ups. The study was approved by the Regional Ethical Committee of South Norway, and

written informed consent was obtained from all participants. The study consists of four waves. New participants were recruited at waves 1 and 3. Participants were scanned up to 11.1 years after the initial scan. The interval between waves was approximately 3.5, 4.4 and, 1.7 years, respectively. Data acquisition took place between 2006 and 2018. MRI sequences were acquired with a 1.5T Avanto and with 3.0T Skyra and Prisma scanners, though no Prisma data was used here. For more details see [6,8–10]. 1176 observations from 635 participants (mean age = 48.1 ± 18.1; age-range = 20.0 - 89.4; females = 425; nTimepoints = 1-4) were included in the main LCBC sample here. **Constructive Memory**. This project is a cross-sectional study where cognitively healthy adults spanning the whole age-span underwent an fMRI source-item memory task. The protocol also included MRI scanning and neuropsychological evaluation. The sample was collected at the center for LCBC. The project is nested within the Cognition and Plasticity through the Lifespan project. All participants gave written informed consent and the study was approved by the Regional Ethical Committee of South Norway. Data acquisition took place between 2013 and 2015. MRI sequences were acquired with a 3.0TSkyra scanner. See [3,11,12] for more details. 163 observations from 163 participants (mean age = 35.5 ± 14.5; age-range = 20.0 - 76.8; females = 110; nTimepoints = 1) were included in the main LCBC sample here. **Method of Loci**. This study consists of an eight-week memory training experiment focused on improving verbal recall memory by implementing the mnemonic technique "method of loci". Participants were randomly assigned to two groups, either the intervention group or a control group serving as passive controls. Participants were scanned three times: pre and post-training and a follow-up after 5 years. Volunteers were recruited through a local newspaper ad and screened by a structured interview before inclusion. Cognitive assessments and the memory training program were conducted at the center for LCBC. All participants gave informed consent, and the study was approved by the Regional Ethical Committee of South Norway. Data acquisition for waves 1 and 2 took place between 2007 and 2008 while the third wave was acquired in 2013. MRI sequences were acquired with a 1.5T Avanto scanner. See [13,14] for more details. 117 observations from 46 participants (mean age = 63.0 ± 9.2; age-range = 41.9 – 82.6; females = 24; nTimepoints = 1-3) were included in the main LCBC imaging sample here. No observations from this project were included in cognitive analyses here. **Neurocognitive plasticity**. The study consists of an experimental project of memory training with the method of loci. The study includes two groups of participants (young and old) that underwent an ABAB design where a batch in each group started with a resting condition, and the other started with memory training (after a baseline test). The study also includes an active group without memory training. The

participants were scanned 6 times, five of them before/after a block of training while the sixth time point consisted of a follow-up ≈ 2 years after the intervention. All procedures were approved by the Regional Ethical Committee of South Norway, and written consent was obtained from all participants. Participants were recruited through newspaper and web page adverts and were screened with a health interview. Participants were required to be either young or older (in or around their 20s or 70s, respectively) healthy adults. Data acquisition took place between 2013 and 2018. MRI sequences were acquired with a 3.0T Skyra scanner. See [15,16] for more details. 942 observations from 226 participants (mean age = 56.5 ± 22.9; age-range = 20.1 84.0; females = 132; nTimepoints = 1-6) were included in the main LCBC imaging sample here. No observations from this project were included in cognitive analyses here.

MRI preprocessing. First, T1w data was processed in FreeSurfer 6.0 cross-sectionally, which includes steps such as removal of non-brain tissue, Talairach transformation, intensity normalisation, demarcating the grey/white and grey/CSF boundaries, and reconstructing the cortical surface. This automated pipeline yields a reconstructed surface map and cortical thickness estimates for each person at each timepoint. To extract more reliable thickness estimates, the reconstructed images were processed longitudinally. Specifically an unbiased within-subject template space and image is created using robust, inverse consistent registration [17]. Several processing steps, such as skull stripping, Talairach transforms, atlas registration as well as spherical surface maps and parcellations are then initialized with common information from the within-subject template, significantly increasing reliability and statistical power of the cortical thickness estimates [18].

Sample	N unique	N obs	N Longitudinal	Mean Time Interval (SD)	Interval Range	N Timepoints	Mean Age (range)	Sex (F/M)
LCBC (discovery)	1084	2577	1851	2.7(2.8)	0.1 - 11	1-6	50.0(20.0-89.4)	703/381
Cam-CAN	634	898	528	1.4(0.7)	0.2 - 3.5	1-2	55.5(20.2-91.6)	323/311
BASE-II	447	768	642	1.9(0.7)	0.6 - 3.1	1-2	62.4(24.1-83.1)	170/277
BETULA	310	480	340	4(0.2)	3.4 - 4.6	1-2	62.7(25.5-84.8)	159/151
DLBS	471	763	584	3.9(0.4)	2.5 - 5.6	1-2	59.7(20.6-93.1)	292/179

Supplementary Table 1 Description of the discovery sample (LCBC) and four replication samples. Intervals are in years.

			N Timepoints							Mean Clinical Dementia Rating	
Group	N unique	N Obs	2	3	4	Mean Time Interval (SD)	Interval Range	Mean Age (range)	Sex (f/m)	All timepoints	Longitudinal timepoints
NC-long	128	435	22	33	73	2.9 (1.4)	1.0 - 6.5	73 (60.5 - 90.2	221/214	0.02	0.01
AD-long	41	110	20	14	7	2.5 (1.1)	1.3 - 5.3	74.7 (55.1 - 89.3)	55/55	0.8	0.93

Supplementary Table 2 Description of the AIBL Alzheimer's disease (AD) sample groups. Intervals are in years.

Sample	Scanner	Sequence	Tesla	Head coil	Slices	Voxel size (mm)	Time parameters	Other parameters
LCBC (discovery)	Avanto (Siemens)	3D MP-RAGE	1.5	12-channel	160	1.25×1.25×1.25	TR/TE/TI=2400ms/3.61ms/1000ms	FA/FOV = 8°/240×240m
	Skyra (Siemens)	3D MP-RAGE	3	32-channel	176	1×1×1	TR/TE/TI=2300ms/2.98ms/850ms	FA/FOV = 8°/256×256m
Cam-CAN	Tim Trio (Siemens)	3D MP-RAGE	3	32-channel	192	1×1×1	TR/TE/TI=2250ms/2.98ms/900ms	FA/FOV = 9°/256×240m
BASE-II	Tim Trio (Siemens)	3D MP-RAGE	3	32-channel	176	1×1×1	TR/TE/TI=2500ms/4.77ms/1100ms	FA/FOV = 7°/256×256m
BETULA	Discovery (GE)	3D FSPGR	3	32-channel	176	1×1×1	TR/TE/TI=8.19ms/3.2ms/450ms	FA/FOV = 12°/250×250r
DLBS	Achieva (Philips)	3D MP-RAGE	3	8-channel	160	1×1×1	TR/TE/TI=2300ms/8.13ms/1100ms	FA/FOV = 12°/204×256r
AIBL (clinical dementia)	Avanto (Siemens)	3D MPRAGE	1.5	NAV	160	1×1×1.2	TR/TE/TI=2300ms/2.98ms/900ms	FA/FOV = 9°/240×256m
	Verio (Siemens)	3D MPRAGE	3	NAV	160	1×1×1.2	TR/TE/TI=2300ms/2.98ms/900ms	FA/FOV = 9°/240×256m
	TrioTim (Siemens)	3D MPRAGE	3	NAV	160	1×1×1.2	TR/TE/TI=2300ms/2.98ms/900ms	FA/FOV = 9°/240×256m

Supplementary Table 3 MRI acquisition parameters by sample. TR =Repetition time; TE= Echo time; TI = Inversion time; FA = Flip angle; FOV = Field of view; NAV = not available [19]; 3D MPRAGE = three-dimensional magnetization prepared rapid gradient echo; 3D FSPGR = three-dimensional fast spoiled gradient echo.

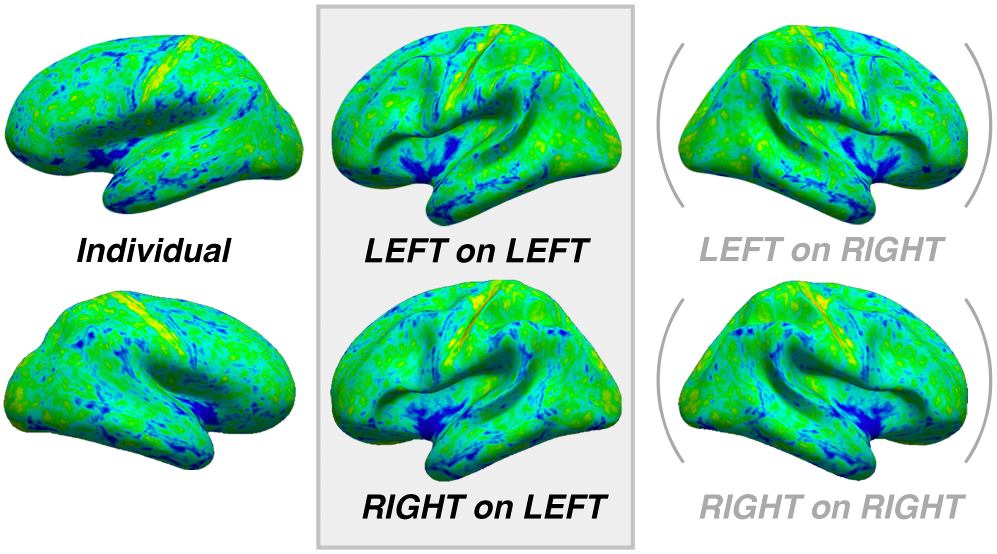

Supplementary Fig. 1. Left and right cortical thickness maps of an example subject shown on the individual surface (see left), and after resampling the data from both hemispheres into homotopic alignment using either one hemisphere of the symmetrical surface. All analyses were performed after resampling to the left symmetrical surface (middle). For visual comparison data is also shown on the right symmetrical surface (right).

Supplementary Note 1

Conventional vertex-wise linear mixed effect (LME) analyses assessing the linear Age × Hemisphere interaction (F-test with Age, Age^2,Age^3) yielded near-identical results to the Age × Hemisphere GAMM interaction (Supplementary Fig.2A; Dice coefficient = 0.84; 96% of significant vertices were encompassed within LME effects), indicating significance results from our non-linear approach were not due to overfitting.

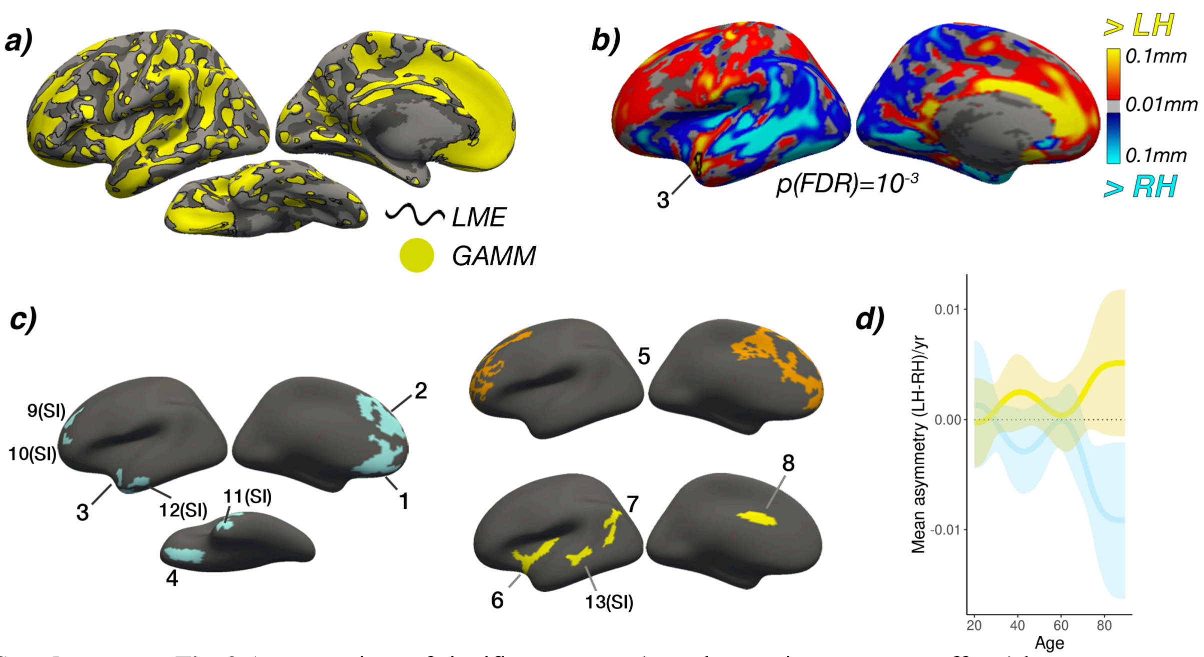

Supplementary Fig. 2 A. comparison of significance maps (age-changes in asymmetry effects) between our non-linear Generalized Additive Mixed Models (GAMM) approach (yellow) compared to a conventional linear mixed model (LME) approach (black outline). 96% of vertices exhibiting significant GAMM effects (one-sided test) were encompassed within vertices showing significant LME effects (results shown uncorrected for multiple comparisons). **B.** The main effect of Hemisphere (as shown in Fig. 2B; derived from GAMMs; two-sided test; False Discovery Rate [FDR]-corrected for multiple comparisons [20]) after resampling to fsaverage5 to permit visual comparison with clustering results. ROI #3 is shown to illustrate the overlap with asymmetry effects in the temporal lobe. **C.** The ROI's derived from clustering (numbered 1-13). The trajectories of ROI's marked with SI are not shown in the main paper but can be seen in Supplementary Fig.4). **D.** The first derivative of the asymmetry trajectories for cluster solutions 1 and 3 in the LCBC discovery sample. The mean timeline of change was highly similar for ROI's exhibiting LH-asymmetry loss and RH-asymmetry loss across the adult lifespan.

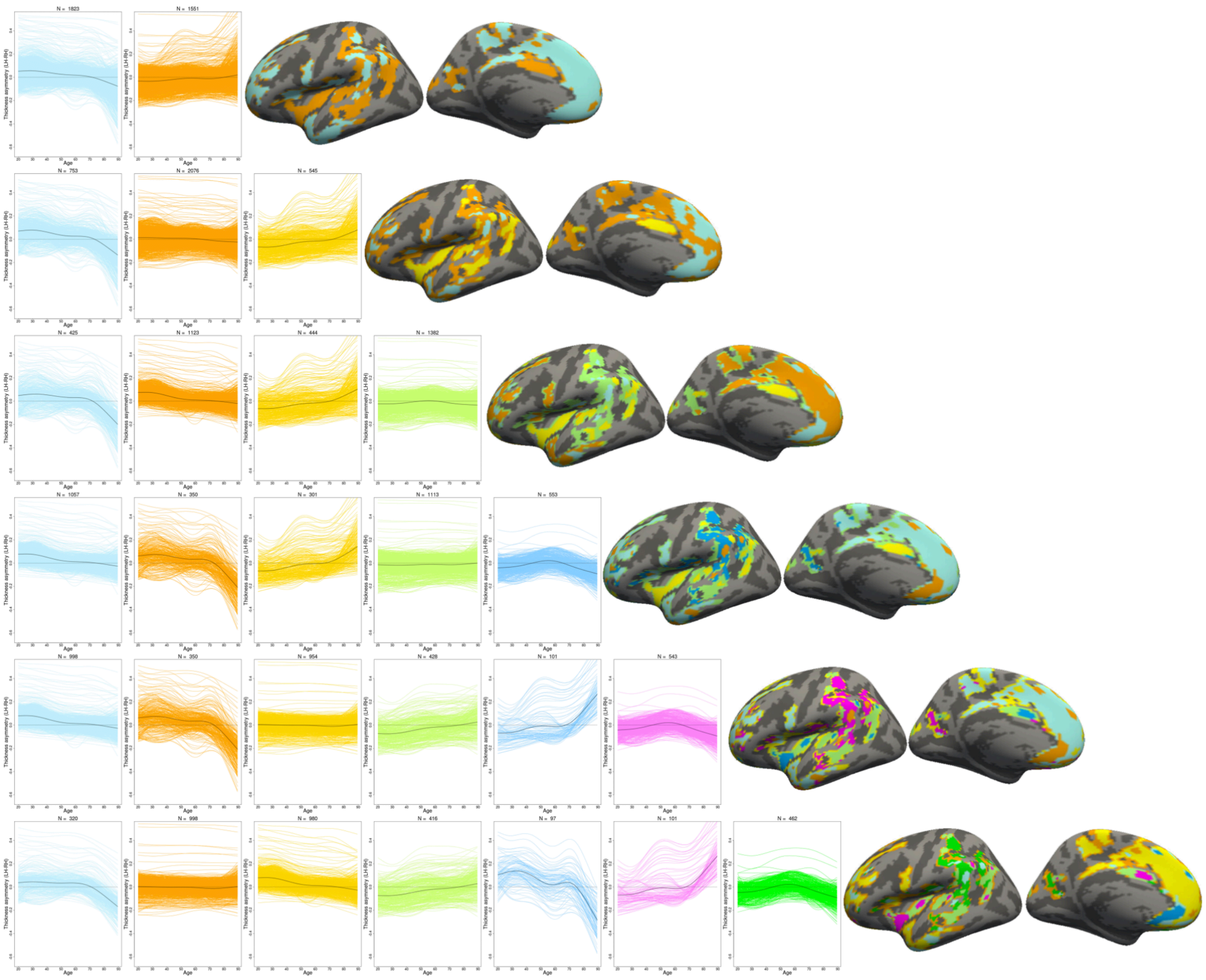

Supplementary Fig. 3 Clustering of asymmetry trajectories (LCBC discovery sample). The main findings were robust to varying the number of PAM clustering partitions. Analyses can be reproduced at https://github.com/jamesmroe/AgeSym.

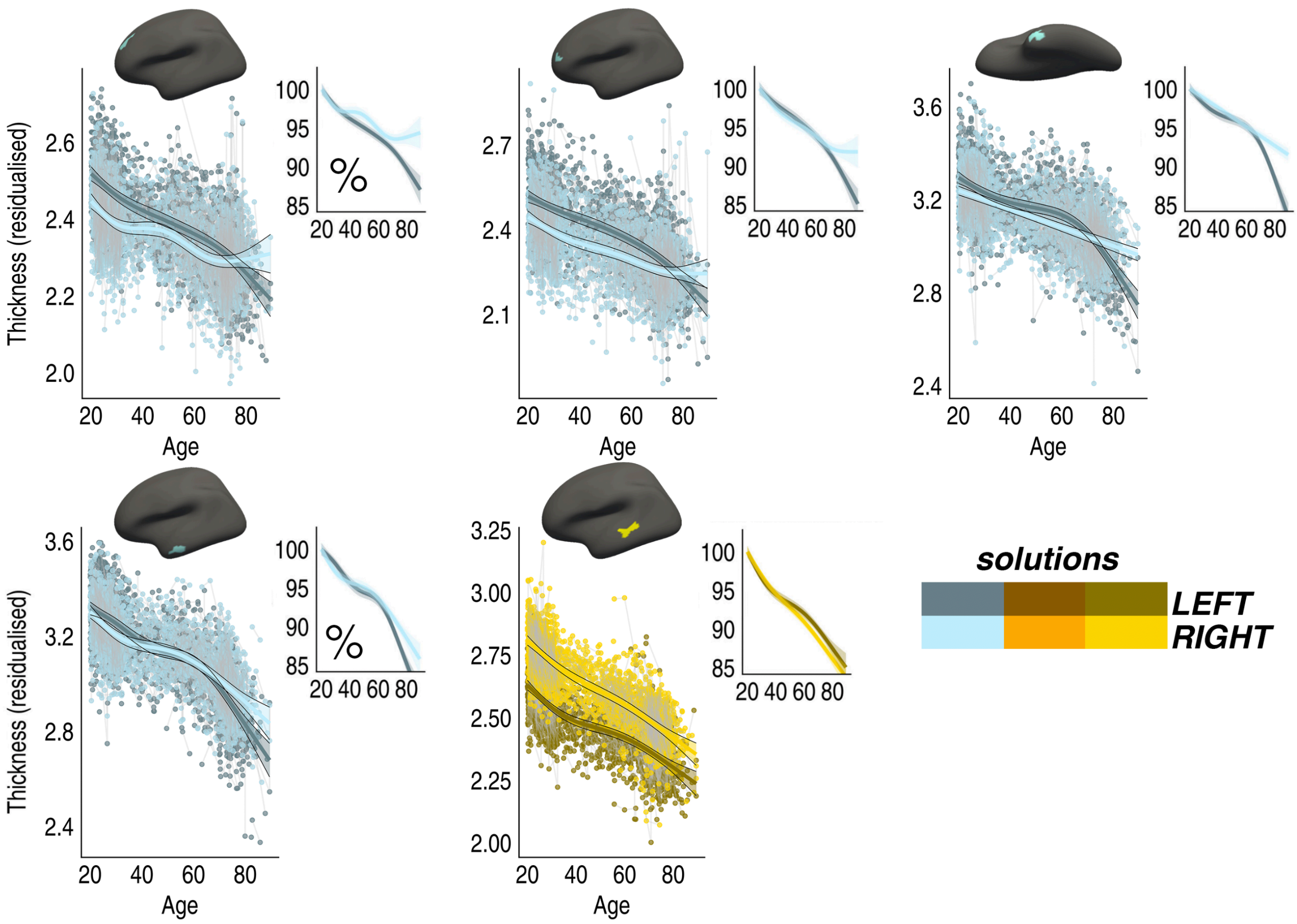

Supplementary Fig. 4 Age-trajectories of cortical thickness plotted separately for the left and right hemisphere for the remaining clustering-derived regions. All trajectories were fitted using Generalized Additive Mixed Models (GAMMs). Colors correspond to the solutions in Fig.3A (main paper), and darker shades indicate left hemisphere trajectories. Data is residualized for sex, scanner and random subject intercepts. Colored trajectories depict mean thickness and ribbons depict 95% confidence intervals. Smaller plots illustrate percentage-change with age for each region. As outliers were detected and removed on a region-wise basis (see Methods), the number of observations underlying plots (clockwise from top left) are: 5152, 5150, 5152, 5150, 5148, respectively.

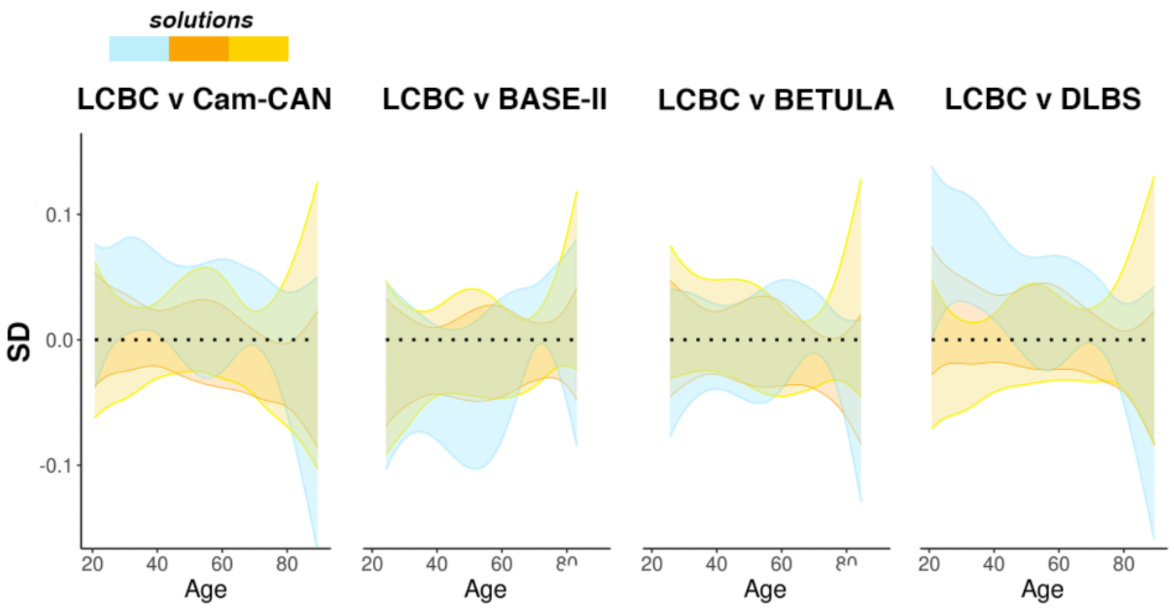

Supplementary Fig. 5 Comparison of the mean asymmetry trajectories between replication and discovery samples for each clustering solution. The means and standard deviations of the three clustering solutions in each sample were subtracted from those in the LCBC discovery sample. Colors correspond to the solutions in Fig. 3 (main paper). Standard deviation was used as the measure is not influenced by the (arbitrary) number of vertices on the surface.

Supplementary Note 2

Vertex-wise GAMM Age × Hemisphere effects appeared largely consistent in Cam-CAN, BASE-II and to a lesser extent BETULA (Supplementary Fig.6). Spatial correlation of effect size maps with LCBC confirmed the impression that vertex-wise significance of age-related change in asymmetry effects replicated in Cam-CAN and BASE-II (respectively r = .45, r = .52), though did not substantiate a replication of vertex-wise effects in BETULA (r = .01) or DLBS (r = .08). The main effect of Hemisphere was highly reproducible across samples (Supplementary Fig. 7).

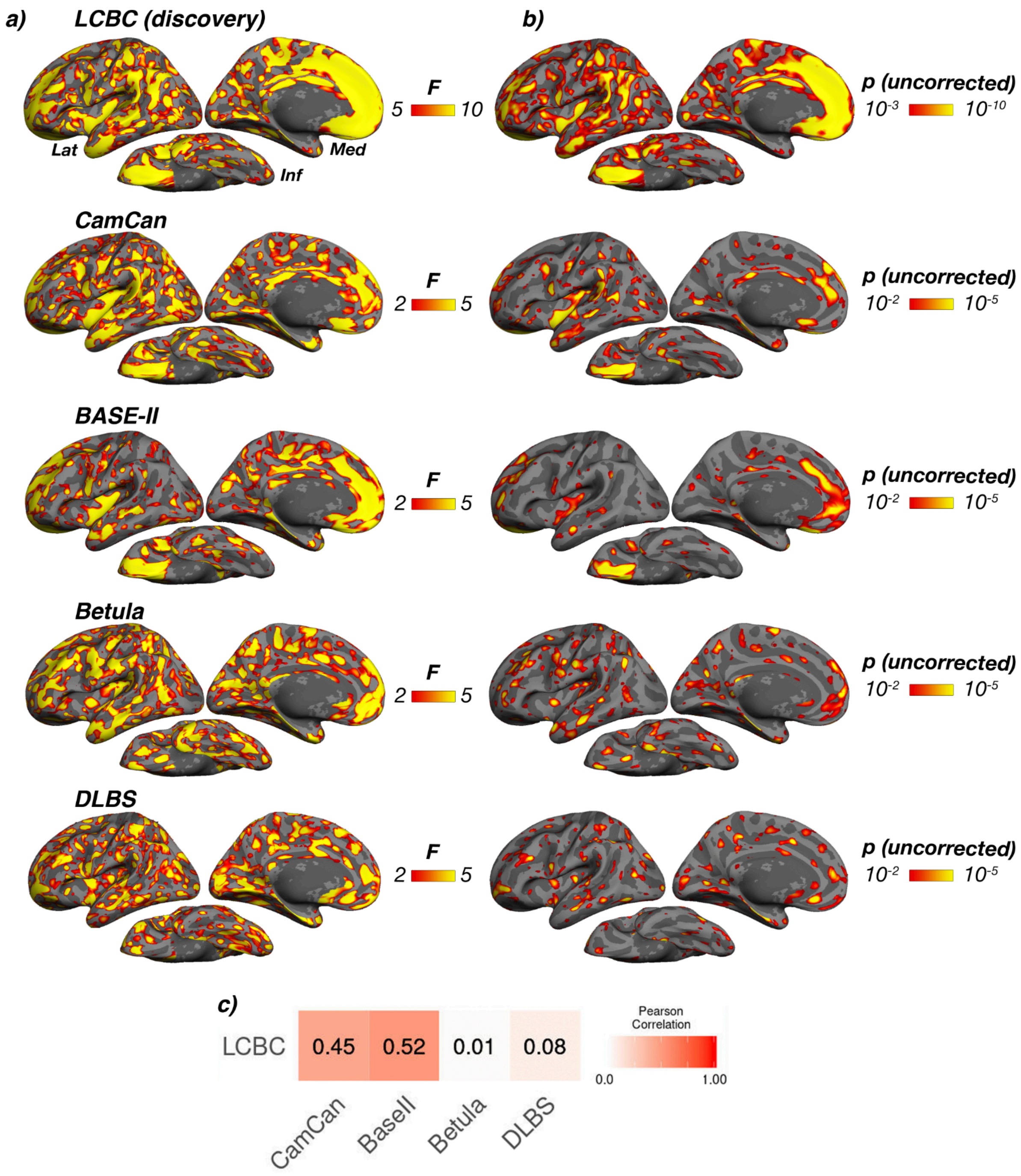

Supplementary Fig. 6. Vertex-wise GAMM age-related change in asymmetry effects for all samples. Samples are depicted row-wise. Note that for visualization purposes, a slightly different visualization threshold is shown for LCBC data to account for differences in sample size/test power. **A)** F values and **B)** significance for the Age × Hemisphere GAMM interaction (one-sided F-test; uncorrected for multiple comparisons). **C)** spatial correlation of effect size maps (Ω^2; maps not shown) for each sample against Ω^2 in the main LCBC discovery sample. Ω^2 was used to assess overlap as the relative values are unaffected by differences in sample size/test power. Spatial correlation between discovery and replication samples was quantified using Pearson's r. Lat = lateral; med = medial; inf = inferior. All maps available at https://github.com/jamesmroe/AgeSym or DOI 10.17605/OSF.IO/XD7CF.

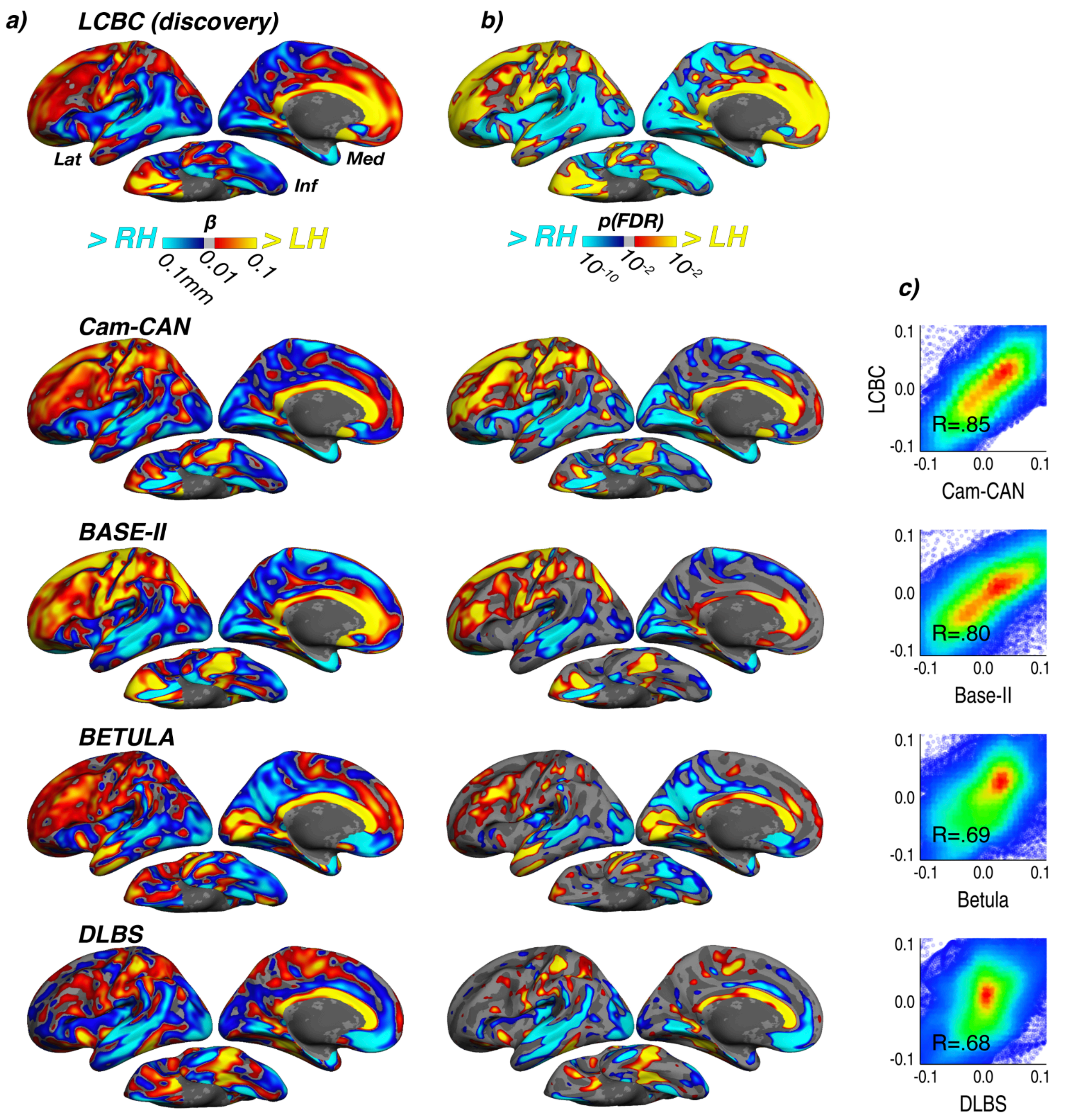

Supplementary Fig. 7. Vertex-wise main effect of Hemisphere for all samples. Samples are depicted row-wise. **A)** Beta coefficients (in mm) and **B)** significance effects (FDR-corrected [20] for multiple comparisons) for the main effect of Hemisphere (GAMM; two-sided test). Note that differences in sample size (and consequently test power) between samples affect the overall level of significance in column B but not A. **C)** spatial correlation of Beta coefficients for each sample against Beta coefficients in the main LCBC discovery sample. Spatial correlation between discovery and replication samples was quantified using Pearson's r. LH = left hemisphere; RH = right hemisphere; Lat = lateral; med = medial; inf = inferior. All maps available at https://github.com/jamesmroe/AgeSym or DOI 10.17605/OSF.IO/XD7CF.

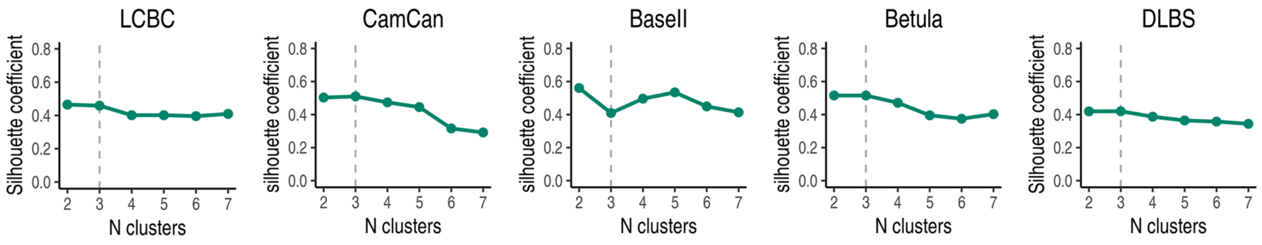

Supplementary Fig. 8 Clustering analysis in replication samples. Mean silhouette width used to determine the optimal number of clustering partitions (N clusters), here shown plotted for each cohort. The chosen 3-cluster solution (identified in the LCBC discovery sample and subsequently applied in replication cohorts) showed stability across samples. Note that BASE-II is the only cohort with non-continuous age sampling which will affect the estimation and subsequent clustering of trajectories (cf. Fig. 1 in main paper).

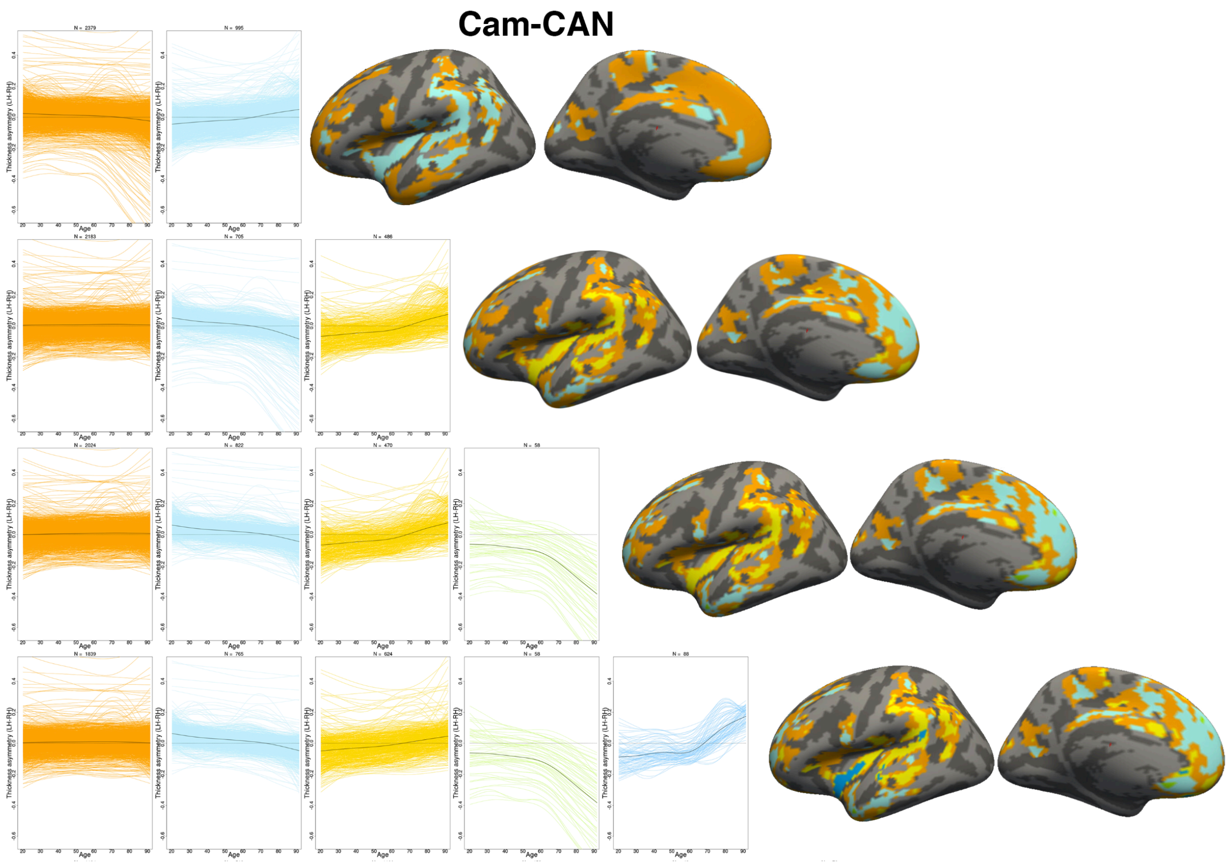

Supplementary Fig. 9 (above and continued below) Clustering analysis in replication samples. The main findings were conserved when varying the number of PAM clustering solutions also in replicating cohorts. Note that colors of the clustering maps are not comparable across cohorts because the order in which clusters are identified by the algorithm will necessarily differ across cohorts. Analyses can be reproduced at https://github.com/jamesmroe/AgeSym or DOI 10.17605/OSF.IO/XD7CF.

Supplementary Fig. 9 (continued)

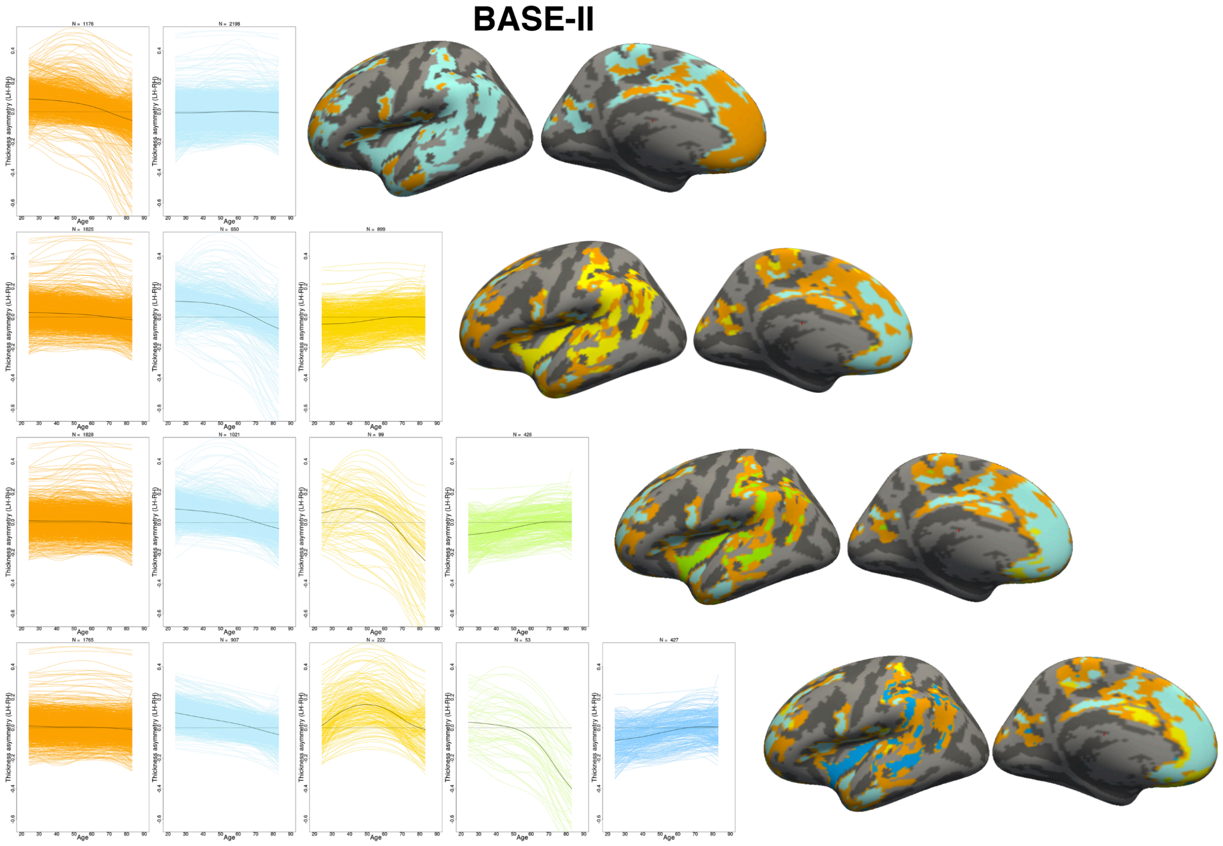

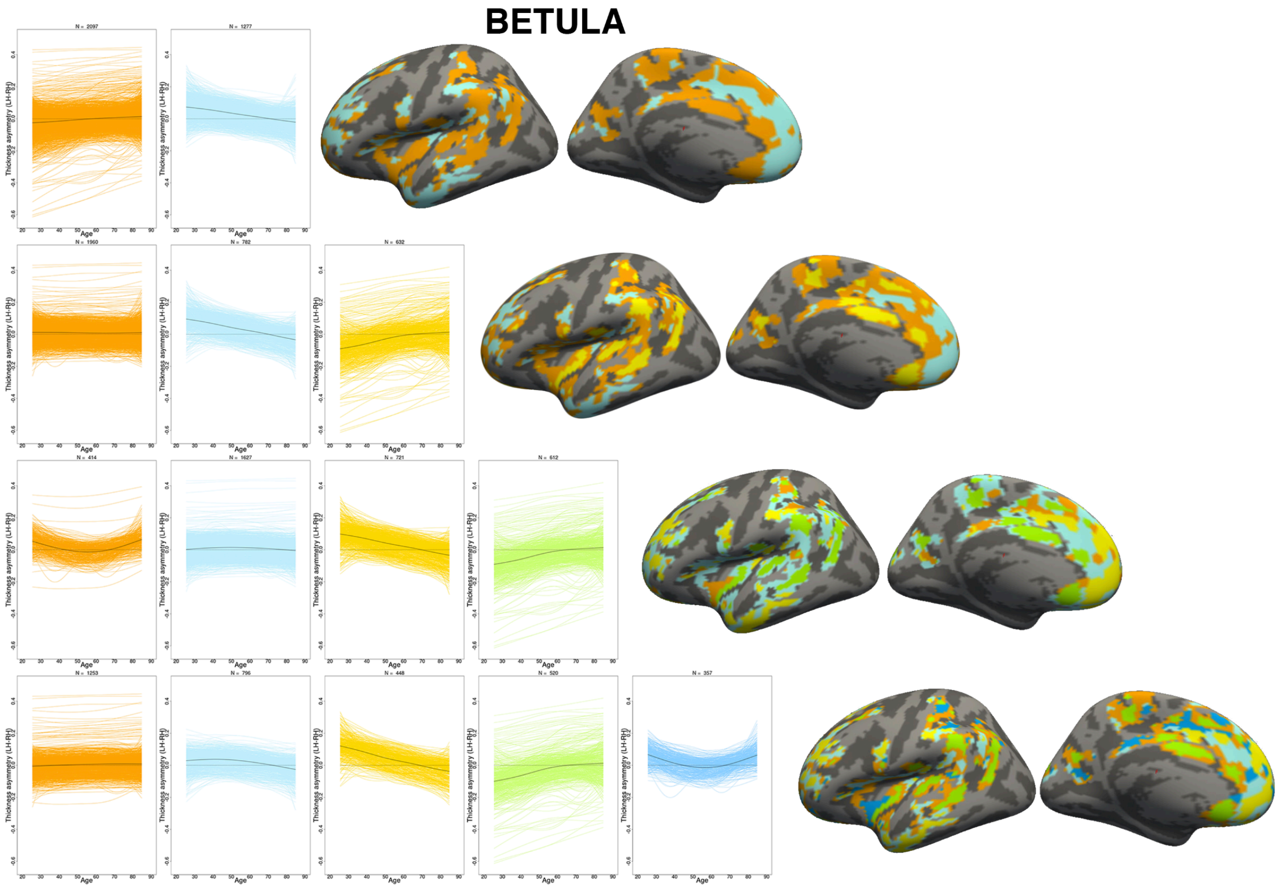

Supplementary Fig. 9 (continued)

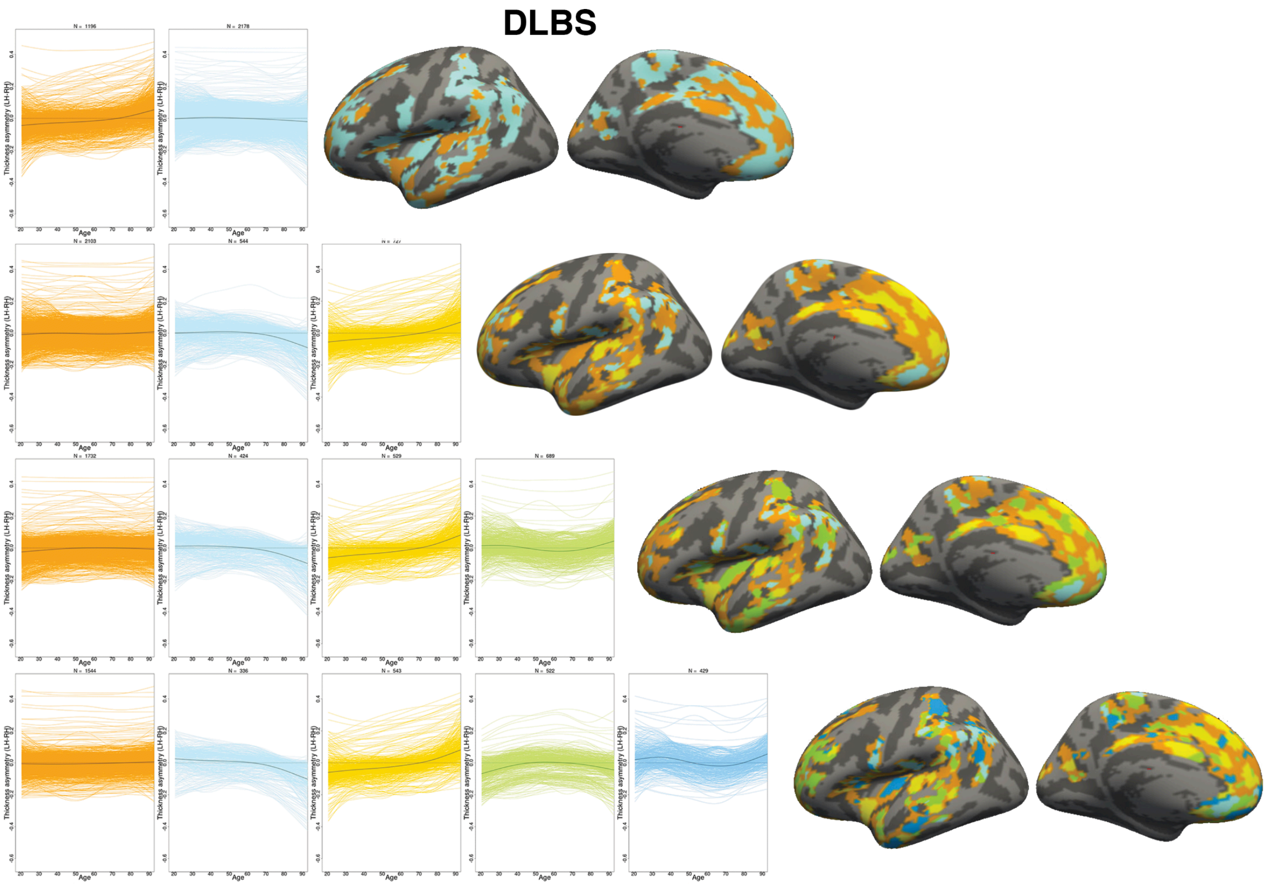

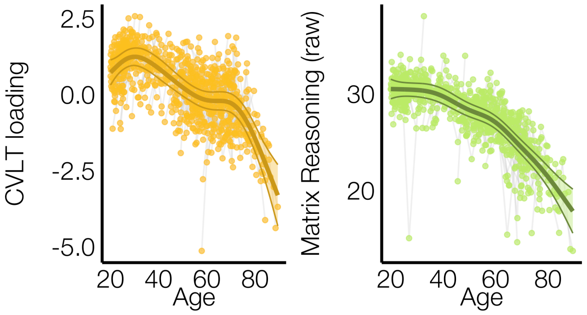

Supplementary Fig. 10 GAMMs revealed that Age (Sex corrected) explained 29% and 36% of the variance in changes in memory (CVLT) and fluid reasoning ability (Matrix reasoning), respectively. Data is residualized for sex, (CVLT) test version and random subject intercepts. See Supplementary Table 4 for full stats.

	**Age effect**			**Covariate**	**Model**	
	edf	*F*	*p-uncorrected*	*Sex (p-uncorrected)*	$r^{2\ (adjusted)}$	Ω^2
CVLT	6.12	25.57	10^{-28}	<0.001	0.29	0.16
Matrix reasoning	4.61	58.45	10^{-49}	0.5	0.36	0.25

Supplementary Table 4 Full GAMM results (one-sided F-tests) predicting changes in verbal memory (CVLT) and fluid reasoning ability (Matrix reasoning) as a function of Age (Sex-corrected). Results are unadjusted for multiple comparisons.

**ROI #**	**Region**	**Clustering solution**	**Cognitive variable**	**Thickness asymmetry (LH-RH)**				**Covariates**			**Model without Age covariate**	
				edf	*F*	*p-uncorrected (Effect direction)*	*p(FDR)*	*Mean thickness (p-uncorrected [effect direction])*	*Sex (p-uncorrected)*	*Scanner (p-uncorrected)*	*Thickness-asymmetry (LH-RH) p-uncorrected (Effect direction)*	*Mean thickness (p-uncorrected [effect direction])*
1	rostral anterior cingulate	1	CVLT	3.34	1.33	0.374	0.72	0.659	4.07E-04	1.34E-03	5.97E-01	2.45E-03 (thicker)
2	superior frontal			2.14	2.11	0.099	0.394	0.355	7.42E-04	4.39E-04	6.91E-01	4.13E-10 (thicker)
3	anterior temporal			1.04	0.37	0.559	0.72	0.042 (thinner)	5.12E-04	1.14E-03	5.38E-02	8.37E-04 (thicker)
4	lateral orbitofrontal			1	5.29	0.022 (lower)	0.291	0.808	1.33E-03	6.18E-04	3.31E-01	1.95E-04 (thicker)
5	frontal cortex	2		1	4.39	0.036 (lower)	0.291	0.648	5.63E-04	2.12E-03	9.70E-01	1.01E-08 (thicker)
6	insula	3		1	0.33	0.565	0.72	0.531	3.39E-04	1.65E-03	1.30E-02 (higher)	7.75E-04 (thicker)
7	inferior parietal			1	0.5	0.479	0.72	0.862	4.39E-04	1.86E-03	3.62E-01	2.43E-05 (thicker)
8	caudal anterior cingulate			1	0.18	0.67	0.731	0.774	3.55E-04	3.15E-03	5.68E-02	4.96E-01
1	rostral anterior cingulate	1	Matrix Reasoning	1	0.3	0.585	0.72	0.094	6.03E-01	6.58E-01	3.01E-02 (higher)	5.59E-10 (thicker)
2	superior frontal			1.54	0.25	0.686	0.731	0.03 (thicker)	3.66E-01	3.29E-01	6.82E-02	1.29E-18 (thicker)
3	anterior temporal			1.92	3.5	0.063	0.336	0.635	6.33E-01	2.97E-01	3.22E-04 (higher)	9.03E-14 (thicker)
4	lateral orbitofrontal			1	0.63	0.427	0.72	0.081	5.44E-01	5.36E-01	1.45E-04 (higher)	5.74E-10 (thicker)
5	frontal cortex	2		1	1.55	0.214	0.654	0.069	4.28E-01	4.42E-01	1.43E-01	1.55E-18 (thicker)
6	insula	3		2.23	0.88	0.32	0.72	0.051	5.58E-01	2.54E-01	8.54E-04 (higher)	3.83E-12 (thicker)
7	inferior parietal			1	0.04	0.845	0.845	0.846	5.17E-01	5.01E-01	2.67E-01	1.67E-09 (thicker)
8	caudal anterior cingulate			2.12	1.4	0.245	0.654	0.026 (thicker)	3.10E-01	9.16E-01	1.59E-01	2.12E-02 (thicker)

Supplementary Table 5 Longitudinal cognitive analyses predicting age-changes in verbal memory (California Verbal Learning Test [CVLT]) and fluid reasoning (Matrix Reasoning subtest of WAIS). GAMM analyses (one-sided F-test) were conducted assessing the predictive value of the smooth term (s) of s(Thickness asymmetry;[LH-RH]) controlling for s(Age), s(Mean thickness; [LH+RH/2]), Sex and Scanner. Results are FDR-corrected for multiple comparisons. No significant effects of Thickness asymmetry were observed after FDR-correction (16 comparisons) in any of the 8 ROI's for either cognitive measure. Estimated degrees of freedom (edf) is an index of curve complexity (i.e. edf = 1 approximates a linear association). P-values pre- and post FDR-correction are shown for Thickness asymmetry effects (columns 7-8). The direction of the asymmetry effect is given for uncorrected p-values < .05 (e.g. higher = more asymmetry relates to better cognition; lower =

less asymmetry relates to better cognition). Effect directions for Mean thickness are also given (thicker = thicker cortex relates to better cognition; thinner = thinner cortex relates to better cognition). Covariate p-values are shown in right columns. Results of comparable GAMM models without covarying for Age are shown in the rightmost columns. Significant asymmetry effects were observed when covarying for mean thickness (across hemispheres) but not age. Uncorrected p-values and directions for the effects of s(Thickness Asymmetry) and Mean thickness are reported. The total number of observations for CVLT and Matrix Reasoning with concurrent scans were 783 (N = 312) and 788 (N = 314), respectively.

Supplementary Note 3

Having observed no significant effects of thickness asymmetry change upon longitudinal cognitive change in any of the 8 ROI's alone, we finally assessed whether regional changes in thickness asymmetry nevertheless contributed a significant combined effect upon cognitive change. Here, we assessed the difference in model fit between a GAMM containing the smooth terms of thickness asymmetry for all 8 ROI's versus one without (covaried for Age, mean thickness [here the first two principal components of the mean thickness of all 8 ROI's], sex and scanner). No significant difference in model fit was observed (verbal memory p = .86; fluid reasoning p = .84), indicating asymmetry had no significant combined effect upon cognitive change.

ROI #	Region	Clustering solution	df	NC-long v AD-long			
				T	*p-uncorrected*	*p(FDR)*	*Sig*
1	rostral anterior cingulate	1	374	-2.34	1.98E-02	4.41E-02	*
2	superior frontal		374	-1.65	9.97E-02	1.59E-01	
3	anterior temporal		374	-3.12	1.95E-03	7.78E-03	*
4	lateral orbitofrontal		374	-4.65	4.65E-06	3.72E-05	*
5	frontal cortex	2	374	-2.3	2.20E-02	4.41E-02	*
6	insula	3	374	-1.02	3.08E-01	4.10E-01	
7	inferior parietal		374	0.83	4.08E-01	4.66E-01	
8	caudal anterior cingulate		374	0.34	7.34E-01	7.34E-01	

Supplementary Table 6 Longitudinal Alzheimer's disease (AD) versus normal controls (NC) analysis. Change-change analyses were conducted using LME's assessing the Group x Years (since baseline) interaction (two-sided test), indicating group differences in thickness asymmetry change over time between longitudinal AD and normal control (NC) groups in clustering-derived ROI's (AD-long; NC-long; see Methods in main paper). Results are corrected for multiple comparisons using FDR. Significant FDR-corrected effects (i.e. p(FDR) < 0.05; *) were observed in frontal cortical and anterior temporal regions. P-values pre- and post FDR correction are shown. AD = Alzheimer's disease; NC = Normal controls; Sig = significance.

Supplementary Note 4

The raw MRI data supporting the results of the current study may be available upon reasonable request, given appropriate ethical, data protection approvals and data sharing agreements. Requests for the raw MRI data can be submitted to the relevant principal investigator of each data contributing study:

Lifebrain Consortium PIs (http://www.lifebrain.uio.no/about/): LCBC (Kristine B. Walhovd – k.b.walhovd@psykologi.uio.no; http://www.oslobrains.no), BETULA (Lars Nyberg – lars.nyberg@umu.se; http://www.ufbi.umu.se/english), BASE-II (Ulman Lindenberger – lindenberger@mpib-berlin.mpg.de; https://www.mpib-berlin.mpg.de/en/research/lifespan-psychology), Cam-CAN (Lorraine K. Tyler – lktyler@csl.psychol.cam.ac.uk; https://cslb.psychol.cam.ac.uk/) (Richard Henson – rik.henson@mrc-cbu.cam.ac.uk; http://www.mrc-cbu.cam.ac.uk/)
Other PI's: Denise Park (DLBS; denise@utdallas.edu; http://agingmind.utdallas.edu/)

AIBL data is available at https://aibl.csiro.au/research/support/ pending application approval and compliance with the data usage agreement.

Supplementary References

1. Tamnes, C. K. *et al.* Brain maturation in adolescence and young adulthood: Regional age-related changes in cortical thickness and white matter volume and microstructure. *Cereb. Cortex* **20**, 534–548 (2010).
2. Fjell, A. M. *et al.* One-Year Brain Atrophy Evident in Healthy Aging. **29**, 15223–15231 (2009).
3. Sneve, M. H. *et al.* Mechanisms Underlying Encoding of Short-Lived Versus Durable Episodic Memories. *J. Neurosci.* **35**, 5202–5212 (2015).
4. Engvig, A. *et al.* Effects of memory training on cortical thickness in the elderly. *Neuroimage* **52**, 1667–1676 (2010).
5. de Lange, A.-M. G. *et al.* White-matter integrity as a marker for cognitive plasticity in aging. *Neurobiol. Aging* **47**, 74–82 (2016).
6. Vidal-Pineiro, D. *et al.* Cellular correlates of cortical thinning throughout the lifespan. Preprint at https://www.biorxiv.org/content/10.1101/585786v3. (2019).
7. Tamnes, C. K. *et al.* Brain development and aging: Overlapping and unique patterns of change. *Neuroimage* **68**, 63–74 (2013).
8. Fjell, A. M. *et al.* The relationship between diffusion tensor imaging and volumetry as measures of white matter properties. *Neuroimage* **42**, 1654–1668 (2008).
9. Storsve, A. B. *et al.* Differential Longitudinal Changes in Cortical Thickness, Surface Area and Volume across the Adult Life Span: Regions of Accelerating and Decelerating Change. *J. Neurosci.* **34**, 8488–98 (2014).
10. Vidal-Piñeiro, D. *et al.* Accelerated longitudinal gray/white matter contrast decline in aging in lightly myelinated cortical regions. *Hum. Brain Mapp.* **37**, 3669–3684 (2016).
11. Vidal-Piñeiro, D. *et al.* Maintained Frontal Activity Underlies High Memory Function Over 8 Years in Aging. *Cereb. Cortex* 1–13 (2018). doi:10.1093/cercor/bhy177
12. Roe, J. M. *et al.* Age-Related Differences in Functional Asymmetry during Memory Retrieval Revisited: No Evidence for Contralateral Overactivation or Compensation. *Cereb. Cortex* **30**, 1129–1147 (2020).
13. Engvig, A. *et al.* Effects of Cognitive Training on Gray Matter Volumes in Memory Clinic Patients with Subjective Memory Impairment. *J. Alzheimers. Dis.* **41**, 779–791 (2014).
14. Engvig, A. *et al.* Memory training impacts short-term changes in aging white matter: A Longitudinal Diffusion Tensor Imaging Study. *Hum. Brain Mapp.* **33**, 2390–2406 (2012).
15. de Lange, A.-M. G. *et al.* The effects of memory training on behavioral and microstructural plasticity in young and older adults. *Hum. Brain Mapp.* **5680**, 5666–5680 (2017).
16. Bråthen, A. C. S. *et al.* Multimodal cortical and hippocampal prediction of episodic-memory plasticity in young and older adults. *Hum. Brain Mapp.* **39**, 4480–4492 (2018).
17. Reuter, M., Rosas, H. D. & Fischl, B. Highly accurate inverse consistent registration: a robust approach. *Neuroimage* **53**, 1181–96 (2010).
18. Reuter, M., Schmansky, N. J., Rosas, H. D. & Fischl, B. Within-subject template estimation for unbiased longitudinal image analysis. *Neuroimage* **61**, 1402–1418 (2012).
19. Ellis, K. A. *et al.* Addressing population aging and Alzheimer's disease through the Australian Imaging Biomarkers and Lifestyle study: Collaboration with the Alzheimer's Disease Neuroimaging Initiative. *Alzheimer's Dement.* **6**, 291–296 (2010).
20. Benjamini, Y. & Yekutieli, D. The control of the false discovery rate in multiple testing under dependency. *Ann. Stat.* **29**, 1165–1188 (2001).
