# Supplementary material for: Asymmetric thinning of the cerebral cortex across the adult lifespan is accelerated in Alzheimer’s disease
Source: Nat Commun. 2021 Feb 1;12:721. doi: 10.1038/s41467-021-21057-y (PMC7851164; doi:10.1038/s41467-021-21057-y)
Supplement: Supplementary file 3 — Reporting Summary [file 41467_2021_21057_MOESM3_ESM.pdf]

nature research

Corresponding author(s): James M Roe

Last updated by author(s): Nov 30, 2020

Reporting Summary

Nature Research wishes to improve the reproducibility of the work that we publish. This form provides structure for consistency and transparency in reporting. For further information on Nature Research policies, see our Editorial Policies and the Editorial Policy Checklist.

Statistics

For all statistical analyses, confirm that the following items are present in the figure legend, table legend, main text, or Methods section.

n/a	Confirmed	
☐	☒	The exact sample size (n) for each experimental group/condition, given as a discrete number and unit of measurement
☐	☒	A statement on whether measurements were taken from distinct samples or whether the same sample was measured repeatedly
☐	☒	The statistical test(s) used AND whether they are one- or two-sided<br>*Only common tests should be described solely by name; describe more complex techniques in the Methods section.*
☐	☒	A description of all covariates tested
☐	☒	A description of any assumptions or corrections, such as tests of normality and adjustment for multiple comparisons
☐	☒	A full description of the statistical parameters including central tendency (e.g. means) or other basic estimates (e.g. regression coefficient) AND variation (e.g. standard deviation) or associated estimates of uncertainty (e.g. confidence intervals)
☐	☒	For null hypothesis testing, the test statistic (e.g. F, t, r) with confidence intervals, effect sizes, degrees of freedom and P value noted<br>*Give P values as exact values whenever suitable.*
☒	☐	For Bayesian analysis, information on the choice of priors and Markov chain Monte Carlo settings
☒	☐	For hierarchical and complex designs, identification of the appropriate level for tests and full reporting of outcomes
☐	☒	Estimates of effect sizes (e.g. Cohen's d, Pearson's r), indicating how they were calculated

Our web collection on statistics for biologists contains articles on many of the points above.

Software and code

Policy information about availability of computer code

Data collection	This is an analysis of previously collected magnetic resonance imaging data from 5 adult lifespan cohorts and 1 clinical dementia cohort. Cohort-specific details for data collection are given in SI Table 2 (MRI scanner and acquisition parameters). For more details see cohort-specific references in Methods. No other software/hardware was used during data collection.
Data analysis	All data analyses were performed using custom scripts written in R (v3.5) and Matlab (2017a). Data preprocessing was performed using FreeSurfer (v6.0). Preprocessing and analysis code is available at https://github.com/jamesmroe/AgeSym

For manuscripts utilizing custom algorithms or software that are central to the research but not yet described in published literature, software must be made available to editors and reviewers. We strongly encourage code deposition in a community repository (e.g. GitHub). See the Nature Research guidelines for submitting code & software for further information.

Data

Policy information about availability of data

All manuscripts must include a data availability statement. This statement should provide the following information, where applicable:

- Accession codes, unique identifiers, or web links for publicly available datasets
- A list of figures that have associated raw data
- A description of any restrictions on data availability

All summary-level surface maps supporting the results are available on the Open Science Framework (OSF; DOI 10.17605/OSF.IO/XD7CF) or at https://github.com/jamesmroe/AgeSym. This data can be used to reproduce all cohort-specific clustering analyses. The raw MRI data may be available upon reasonable request, given appropriate ethical, data protection and data sharing agreements. Requests for the raw MRI data can be submitted to the relevant principal investigator of each data contributing study (https://www.lifebrain.uio.no/). Contact details are provided in Supplementary Notes. Individual-level data availability for some of the samples is restricted as participants have not consented to publicly share their data, and different restrictions apply to different samples. LH_Sym is available at

https://www.gin.cnrs.fr/en/tools/lh-sym/. AIBL data is available at https://aibl.csiro.au/research/support/ pending application approval and compliance with the data usage agreement.

Field-specific reporting

Please select the one below that is the best fit for your research. If you are not sure, read the appropriate sections before making your selection.

- [x] Life sciences
- [ ] Behavioural & social sciences
- [ ] Ecological, evolutionary & environmental sciences

For a reference copy of the document with all sections, see nature.com/documents/nr-reporting-summary-flat.pdf

Life sciences study design

All studies must disclose on these points even when the disclosure is negative.

Sample size	For all analyses, sample size was determined based on data availability. No analyses were performed to predetermine sample sizes. We gathered as much data as we could, from both cross-sectional and longitudinal observations. All MRI scans that did not fail FreeSurfer processing from each site were included.<br>For analyses using AIBL data, as we were specifically interested in quantifying group differences in change, we used only longitudinal observations. All available observations of non-reverting individuals diagnosed with Alzheimer's disease (AD) by their final timepoint, or classified as cognitively healthy throughout, were included in the analysis.
Data exclusions	LCBC discovery sample exclusion criteria were pre-established: participants were required to score <21 on the Beck Depression Inventory and ≥25 on the Mini Mental Status Exam. Based on these criteria, 13 observations were excluded from the initial sample, bringing the total number of observations to 2577.<br>No exclusion criteria was applied to replication datasets.<br>AIBL exclusion criteria were pre-established: AIBL participants reverting from an AD or Mild Cognitive Impairment diagnosis at any later timepoint were excluded to increase the validity of our longitudinally-defined groups.
Replication	We sought replication in 4 independent longitudinal aging cohorts. Results showed full replication in 3 cohorts and partial replication in 1 cohort. Analysis of an independent Alzheimer's disease sample yielded similar results to the healthy aging samples.
Randomization	For the main analysis, randomization is not applicable as there was no group allocation.<br>AIBL participants were assigned to groups based on diagnosis at their final available timepoint. Covariates such as age, sex and site were controlled for.
Blinding	For the main analysis, blinding is not applicable as there was no group allocation.<br>AIBL participant clinical status was decided by a clinical review panel. Blinding may not be applicable for the present study, as the groups tested were defined by longitudinally-derived diagnoses.

Reporting for specific materials, systems and methods

We require information from authors about some types of materials, experimental systems and methods used in many studies. Here, indicate whether each material, system or method listed is relevant to your study. If you are not sure if a list item applies to your research, read the appropriate section before selecting a response.

Materials & experimental systems

n/a	Involved in the study	
x		Antibodies
x		Eukaryotic cell lines
x		Palaeontology and archaeology
x		Animals and other organisms
	x	Human research participants
x		Clinical data
x		Dual use research of concern

Methods

n/a	Involved in the study	
x		ChIP-seq
x		Flow cytometry
	x	MRI-based neuroimaging

Human research participants

Policy information about studies involving human research participants

Population characteristics	The main discovery sample consisted of 2577 scans (1851 longitudinal) from 1084 healthy individuals aged 20.0 to 89.4 (mean age=50.0; 703 females) from the Center for Lifespan Changes in Brain and Cognition database. 1 to 6 timepoints were available per subject.<br>Replication samples consisted of up to 2 timepoints from:<br>Cam-CAN: 898 observations of 634 unique participants (age range=20-91; mean age=55.5; females=323)<br>BASE-II: 768 observations of 447 unique participants (age range=24-83; mean age=62.4; females=170)<br>BETULA: 480 observations of 310 unique participants (age range=25-84; mean age=62.7; females=159)<br>DLBS: 763 observations of 471 unique participants (age range=20-93; mean age=59.7; females=292)<br>AIBL sample consisted of up to 4 timepoints from:<br>NC group: 435 observations of 128 unique participants (age range=60-90; mean age=73; females=221)<br>AD group: 110 observations of 41 unique participants (age range=55-89; mean age=74.7; females=55)
Recruitment	LCBC participants were recruited via newspaper and social media advertisements, and are thus not representative of the population due to non-random sampling. Follow-up observations suffer to some degree from selective attrition as returning participants tend to be healthier and show higher cognitive performance. Overall, the study population tends to be higher educated and perform higher relative to same-age peers. However, this is stable over the whole age-range, and thus it is unlikely to affect the main results and conclusions of the study (i.e. Age × Hemisphere and main effect of Hemisphere), which showed high consistency across independent samples, also in studies employing random recruitment from the population (i.e. Cam-Can and Betula; see associated references in Methods). Selective attrition biases also seem to affect the different ages to a similar degree in the LCBC sample, though it is not unreasonable that older adults are somewhat more affected (due to death, illness, dementia, etc.) in other cohorts. Potentially, this could lead to an underestimation of the loss of asymmetry as only healthier older individuals come for additional follow-ups. This could be one candidate explanation for the lack of full replication of the lifespan trajectories in DLBS. It is also possible that the lack of significant effects we observed for regional changes in thickness asymmetry upon longitudinal cognitive scores may be somewhat affected by the high number of cognitively above-average participants in the LCBC sample. Potential biases and limitations associated with sample recruitment are discussed in the manuscript.<br><br>For specific details of participant recruitment in each longitudinal replication sample, see the cohort-specific reference in Methods.
Ethics oversight	All LCBC studies were approved by the Norwegian Regional Committee for Medical and Health Research Ethics. For ethical approvals of sub-studies, see cohort-specific references in Methods.

Note that full information on the approval of the study protocol must also be provided in the manuscript.

Magnetic resonance imaging

Experimental design

Design type	T1-weighted anatomical scans
Design specifications	This field is not applicable as no experiment was conducted in the present study
Behavioral performance measures	No task was performed in the scanner (only anatomical scans used). For cognitive analyses, we used scores on the California Verbal Learning Test (CVLT) and Matrix Reasoning subtest of Weschler Abbreviated Scale of Intelligence (WASI), acquired as part of a standard neuropsychological test battery

Acquisition

Imaging type(s)	Structural
Field strength	3T and 1.5T
Sequence & imaging parameters	LCBC (Siemens Avanto); 3D MP-RAGE; 1.5 Tesla; 160 slices; 1.25×1.25×1.25 voxel size; TR/TE/TI=2400ms/3.61ms/1000ms ; FA/FOV = 8°/240×240mm<br>LCBC (Siemens Skyra); 3D MP-RAGE; 3 Tesla; 176 slices; 1×1×1 voxel size; TR/TE/TI=2300ms/2.98ms/850ms ; FA/FOV = 8°/256×256mm<br>Cam-CAN (Siemens Tim Trio); 3D MP-RAGE; 3 Tesla; 192 slices; 1×1×1 voxel size; TR/TE/TI=2250ms/2.98ms/900ms ; FA/FOV = 9°/256×240mm<br>BASE-II (Siemens Tim Trio); 3D MP-RAGE; 3 Tesla; 176 slices; 1×1×1 voxel size; TR/TE/TI=2500ms/4.77ms/1100ms ; FA/FOV = 7°/256×256mm<br>BETULA (GE Discovery); 3D FSPGR; 3 Tesla; 176 slices; 1×1×1 voxel size; TR/TE/TI=8.19ms/3.2ms/450ms ; FA/FOV = 12°/250×250mm<br>DLBS (Philips Achieva); 3D MP-RAGE; 3 Tesla; 160 slices; 1×1×1 voxel size; TR/TE/TI=2300ms/8.13ms/1100ms; FA/FOV = 12°/204×256mm<br>AIBL (Siemens Avanto); 3D MPRAGE; 1.5 Tesla; 160 slices; 1×1×1.2 voxel size; TR/TE/TI=2300ms/2.98ms/900ms ; FA/

FOV = 9°/240×256mm
AIBL (Siemens Verio); 3D MPRAGE; 3 Tesla; 160 slices; 1×1×1.2 voxel size; TR/TE/TI=2300ms/2.98ms/900ms ; FA/FOV = 9°/240×256mm
AIBL (Siemens TrioTim); 3D MPRAGE; 3 Tesla; 160 slices; 1×1×1.2 voxel size; TR/TE/TI=2300ms/2.98ms/900ms ; FA/FOV = 9°/240×256mm

Area of acquisition: Whole brain

Diffusion MRI: ☐ Used ☒ Not used

Preprocessing

Preprocessing software: Cortical reconstruction was performed with FreeSurfer's longitudinal pipeline (v6.0.0)

Normalization: We used standard procedures as implemented in FreeSurfer recon-all.

Normalization template: A symmetrical surface template (LH_Sym) was used to resample the FreeSurfer-estimated cortical thickness maps of the left and right hemispheres of each participant into a common analysis space. LH_Sym was created from a composite of LH and RH surface models in a database enriched in left-handers: the BIL&GIN (https://www.gin.cnrs.fr/en/tools/lh-sym/)

Noise and artifact removal: Standard recon-all procedures were used. Only scans failing reconstruction were not included. We employed a statistical approach to identify potentially poorer quality reconstructions by identifying outliers >6 SD from the fitted trajectory of either hemisphere in our clustering-derived ROI's, and removing these data points from statistical analysis on a region-wise basis.

Volume censoring: No volume censoring was performed. For the LCBC sample, all images were checked for motion artefacts at scan acquisition, and a repeated scan was taken if high motion was evident.

Statistical modeling & inference

Model type and settings: We used Generalized Additive Mixed Models (GAMMs) and a factor-smooth GAMM interaction approach to assess the smooth non-linear Age × Hemisphere interaction across the cortex. Hemisphere, Sex and Scanner were included as fixed effects, and a random subject intercept was included.

Effect(s) tested: Age × Hemisphere interaction as assessed by a factor-smooth GAMM approach.
Main effect of Hemisphere

Specify type of analysis: ☒ Whole brain ☐ ROI-based ☐ Both

Statistic type for inference (See Eklund et al. 2016): Vertex-wise

Correction: FDR correction for positive dependency (Benjamini and Yekutieli method).

Models & analysis

n/a	Involved in the study
☒	☐ Functional and/or effective connectivity
☒	☐ Graph analysis
☒	☐ Multivariate modeling or predictive analysis
